# A novel target state detection method for accurate cardiopulmonary signal extraction based on FMCW radar signals

**DOI:** 10.3389/fphys.2023.1206471

**Published:** 2023-06-26

**Authors:** Xiaozheng Zhang, Chenxi Yang, Zhengyu Xiao, Binbin Lu, Ji Zhang, Jianqing Li, Chengyu Liu

**Affiliations:** ^1^ The State Key Laboratory of Digital Medical Engineering, School of Instrument Science and Engineering, Southeast University, Nanjing, China; ^2^ Chuhang Technology Co. Ltd., Nanjing, China

**Keywords:** FMCW radar, target state detection, range bin, cardiopulmonary signal, respiration rate, heart rate

## Abstract

Frequency-modulated continuous wave radar is capable of constant, real-time detection of human presence and monitoring of cardiopulmonary signals such as respiration and heartbeat. In highly cluttered environments or when the human body moves randomly, noise signals may be relatively large in some range bins, making it crucial to accurately select the range bin containing the target cardiopulmonary signal. In this paper, we propose a target range bin selection algorithm based on a mixed-modal information threshold. We introduce a confidence value in the frequency domain to determine the state of the human target and employ the range bin variance in the time domain to determine the range bin change status of the target. The proposed method accurately detects the state of the target and effectively selects the range bin containing the cardiopulmonary signal with a high signal-to-noise ratio. Experimental results demonstrate that the proposed method achieves better accuracy in cardiopulmonary signal rate estimation. Moreover, the proposed algorithm is lightweight in data processing and has good real-time performance.

## 1 Introduction

Respiration and heartbeat can indicate the basic physiological health status of the human body and are also adopted for clinical diagnosis (Gu, 2016; Weenk et al., 2017). However, traditional vital sign detection methods, such as electrocardiography or photoplethysmography, are contact-based and require electrodes to be in contact with the body surface, which can cause discomfort with long-term use (Castaneda et al., 2018; Kebe et al., 2020). Non-contact radar for vital sign detection is a promising approach that offers increased flexibility in usage. Radars can be placed anywhere in a given space and provide continuous monitoring without the patient’s perception (Morgan and Zierdt, 2009; Obeid et al., 2012; Will et al., 2016; Yang et al., 2020). It overcomes the discomfort of the long-term use of electrodes in wearable sensors and is of great value to newborn babies, burned patients, and infectious disease patients in hospitals (Zhao et al., 2013). The remote monitoring of vital signs of the radar system is more and more widely used. Radar can detect human presence in indoor environments (Mercuri et al., 2013; Gennarelli et al., 2022). Radar can track the periodic movement of body parts caused by breathing, which can be used for system diagnosis of breathing problems such as apnea (Sun, 2019; Islam et al., 2021; Ishrak et al., 2023). Radar sensing technology can also perform gesture recognition and classification for human-computer interaction (Sharma et al., 2023). The latest research shows that radar systems can also accurately extract cardiac physiological parameters for estimating heartbeat intervals and cardiac timing (Xia et al., 2021). Its application scenarios also incorporate the detection of diseases such as sleep (Hong et al., 2018); monitoring of the driver’s health status (Park et al., 2019; Wang et al., 2021); earthquake rescue detection (Liu et al., 2011), etc.

The radars currently used for vital signs detection include frequency-modulated continuous wave (FMCW) radar (He et al., 2017; Ahmad et al., 2018), ultra-wideband (UWB) radar (Deng et al., 2019; Yang et al., 2021; Sharma et al., 2023), continuous wave (CW) radar (Hong et al., 2018; Gennarelli et al., 2022), etc. The FMCW radar has a wide range of applications for continuous non-contact vital sign monitoring in a variety of scenarios. It provides high-ranging accuracy and can detect micro-displacement of the abdomen and chest caused by cardiopulmonary activity. Low-cost low-power radar sensors have advantages in continuous time cardiopulmonary signal monitoring (Suijker et al., 2014). The radar intermediate frequency (IF) signal undergoes Fast Fourier Transform (FFT) to obtain micro-displacement information in each range bin. For cardiopulmonary signal monitoring, it is necessary to select the range bin where the target is located to extract the micro-displacement signal as the cardiopulmonary signal (He et al., 2017; Lee et al., 2019). Then, the respiration rate (RR) and heart rate (HR) are extracted separately from the cardiopulmonary signal. Therefore, the accuracy of respiration rate and heart rate estimation can be improved from two aspects. One is the precise selection of range bins to obtain cardiopulmonary signals with high signal-to-noise ratio (SNR). The other is the separation algorithm of heartbeat and respiration, which eliminates more noise signals.

Firstly, the accurate selection of the range bin is crucial to extract the cardiopulmonary signal with high SNR. The traditional range bin selection method is based on the maximum amplitude or maximum phase (Li et al., 2013; Van Loon et al., 2016; Alizadeh et al., 2019; Sacco et al., 2020), which cannot avoid the large noise caused by the random motion of the human body or other small displacements, so the accuracy of the cardiopulmonary signal may be affected. In (Choi et al., 2020), the selection method based on the magnitude-phase coherence index improves the range bin selection accuracy, but it requires a lot of computing power and lacks real-time evaluation performance.

Then, various signal processing algorithms are employed for heartbeat and respiration signal separation, each with its advantages and limitations (Singh et al., 2020). The most straightforward way to extract RR and HR is to use an FFT on a segment of the phase signal and find the peaks in the spectrum. However, the leakage problem due to the limited data length of FFT leads to degraded detection performance. Both the FFT algorithm and the autocorrelation function are applied to the received signal to extract the cardiopulmonary signal (Chioukh et al., 2011). In (Lee et al., 2016), the authors addressed the FFT smearing and leakage problem by using the multiple signal classification algorithm. In (Lv et al., 2021), an algorithm combining complete ensemble empirical mode decomposition with adaptive noise and fast independent component analysis was employed to investigate short-term heart rate measurement techniques. In order to obtain higher estimation accuracy, these methods increase the complexity of the algorithm and require more computing power.

There are currently few research algorithms for cardiorespiratory monitoring at the low sampling rate of low-power radars. To meet the real-time and lightweight requirements of the algorithm, this paper proposes a target state detection method based on mixed-modal thresholds that accurately selects the target range bin and extracts cardiopulmonary signals with high SNR. In the frequency domain, the target energy confidence value is compared to a threshold to precisely identify the target state. In the time domain, the range bin variance is compared to a threshold to select the optimal range bin in each data frame. For respiration rate and heart rate estimation, this paper utilizes the FFT algorithm, the most commonly used spectral analysis method. While assuming a static human body relative to the radar, this approach provides acceptable accuracy and requires minimal computing power, making it suitable for embedded systems with low-power radars. Furthermore, the proposed method demonstrates robust performance even with lower sampling rate data. The main contributions of this work are summarized as the following:

The proposed method performs well for human target state detection and can select range bins containing cardiopulmonary signals with high SNR. We estimate respiration rate and heart rate with the FFT method. The results demonstrate that the proposed method is more accurate than traditional range bin localization methods.

The proposed method only needs lightweight calculations and has certain adaptability and real-time performance under low sampling rate data, which is extremely valuable for realizing low-cost low-power radar chip embedded systems.

The rest of this paper is organized as follows. Methods are introduced in Section 2. The experimental results are described in detail in Section 3. In Section 4, the conclusion is drawn.

## 2 Methods

**FIGURE 1:**
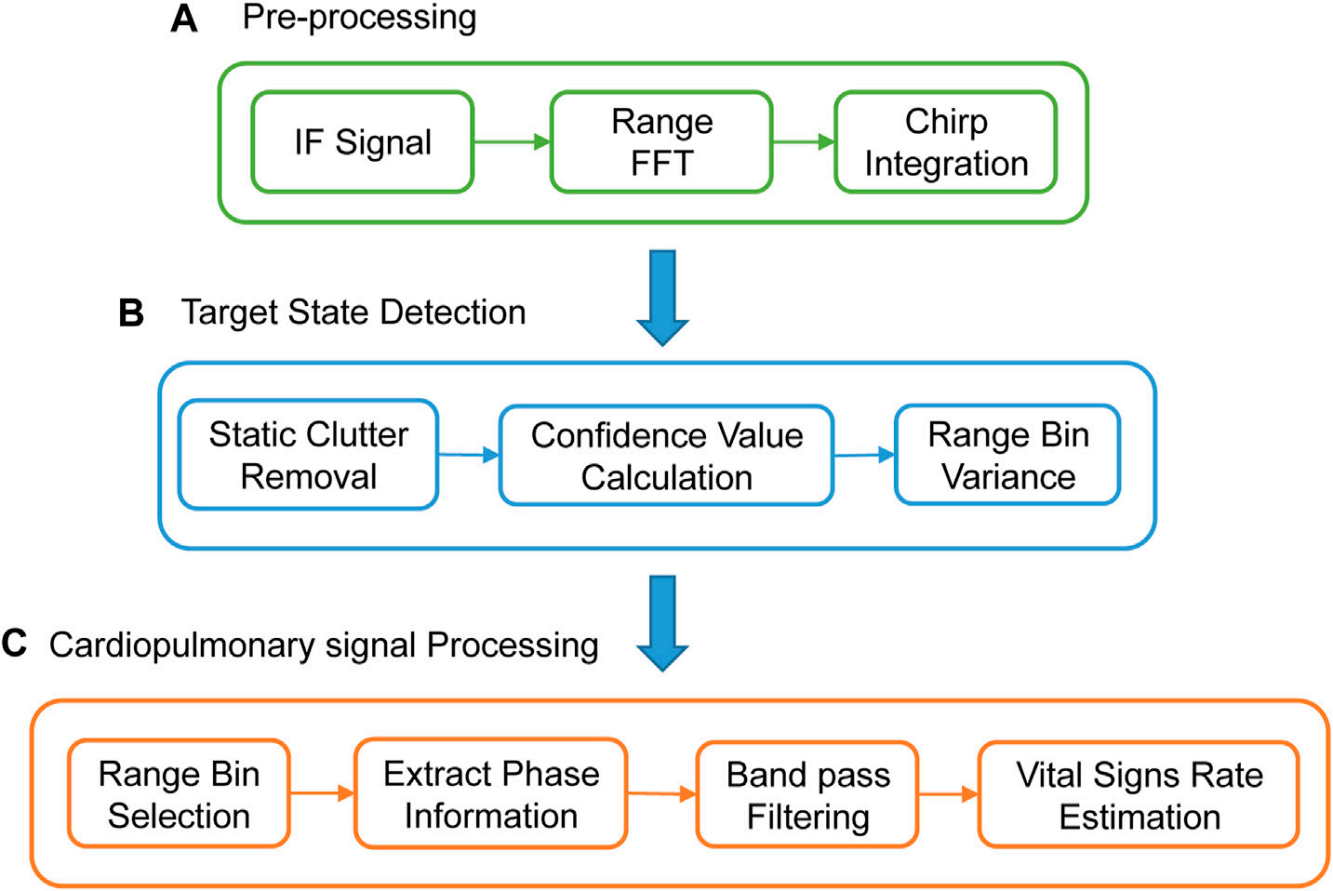
The overall signal processing flow. **(A)** Pre-processing. **(B)** Target state detection flow. **(C)** Cardiopulmonary signal processing.

The process of radar signal processing is presented in Figure 1, which consists of three parts: signal pre-processing, target state detection, and cardiopulmonary signal processing. The innovative part of our proposed algorithm is mainly the target state detection part.

### 2.1 FMCW radar signal model in cardiopulmonary measurements

**FIGURE 2:**
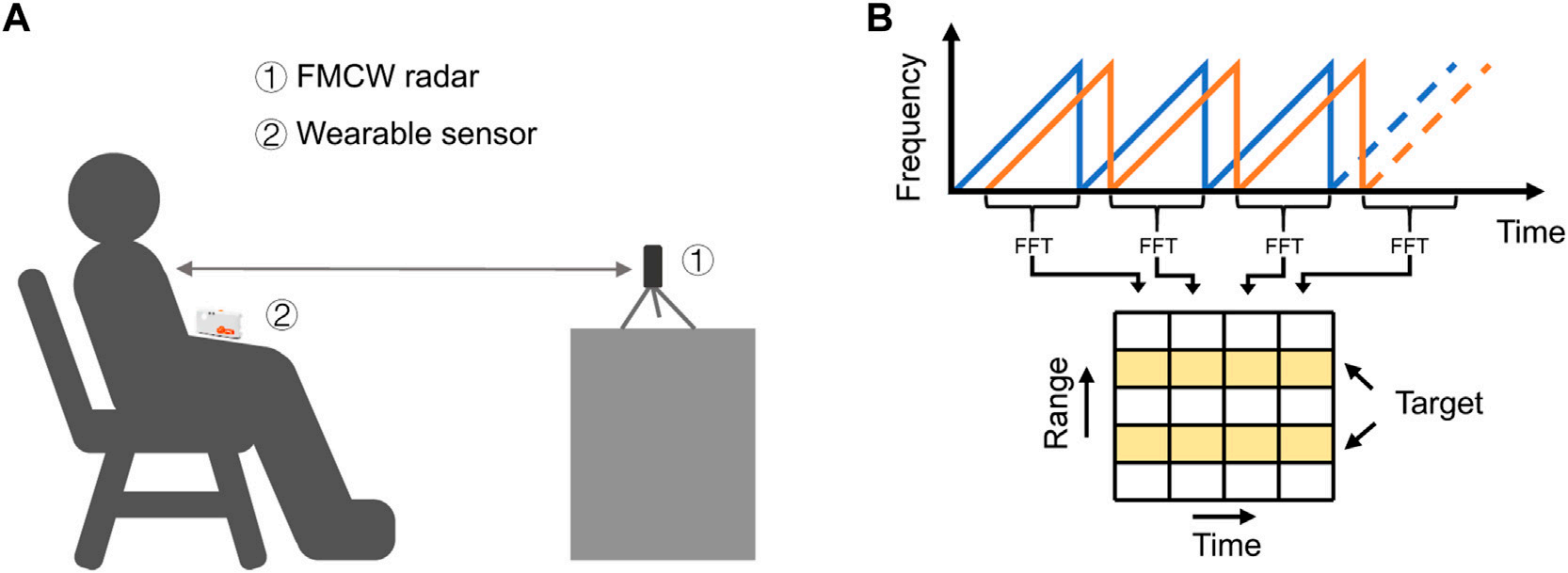
Radar cardiopulmonary monitoring. **(A)** Experimental setup. **(B)** FMCW radar signal model.

Any object in the radar path will reflect the echo signal, and the radar system can detect the distance, speed, and angle of the target by capturing and processing the reflected signal. The experimental setup for radar cardiopulmonary signal monitoring is presented in Figure 2A. Assume that in a chirp period $T_{c}$ , the frequency of the radar transmit signal $f\left(t\right)$ and the transmit wave signal $s_{T}\left(t\right)$ are denoted as:

$$f\left(t\right)=f_{c}+\gamma t,0\leq t<T_{c}$$ (1)

$$s_{T}\left(t\right)=A_{T}\exp\left(j2\pi \left(f_{c}t+\gamma t^{2}/2\right)\right),0\leq t<T_{c}$$ (2)

where $f_{c}$ is the carrier frequency, γ is the slope of the chirp and indicates the change rate of the modulation frequency over time, t is the sweep time, $T_{c}$ is the chirp period, $A_{T}$ is the transmit signal power, and j is an imaginary unit. The radar received signal $s_{R}\left(t\right)$ corresponds to a delay in the transmit signal of time τ can be described as (Alizadeh et al., 2019):

$$s_{R}\left(t\right)=A_{R}\exp\left(j2\pi \left(f_{c}\left(t-\tau \right)+\gamma {\left(t-\tau \right)}^{2}/2\right)\right),\tau \leq t<T_{c}$$ (3)

$$\tau =2\left(R_{0}+x\left(t\right)\right)/c$$ (4)

where $A_{R}$ represents the received signal power, τ is the received time delay, $R_{0}$ stands for the initial distance when the human body remains relatively stationary from the radar, $x\left(t\right)$ is the micro-displacement of the body surface caused by respiration and heartbeat, and c is the speed of light. The transmit signal and the receive signal overlap on $\left[\tau ,T_{c}\right]$ , and the IF signal $s_{IF}\left(t\right)$ is obtained by inputting these two signals into the mixer. The definition of $s_{IF}\left(t\right)$ is expressed as:

$$s_{IF}\left(t\right)=s_{T}\left(t\right)s_{R}^{*}\left(t\right)\approx A_{T}A_{R}\exp\left(j2\pi \left(\gamma \tau t+f_{c}\tau \right)\right)=A_{T}A_{R}\exp\left(j\left(2\pi f_{IF}t+\varphi \left(t\right)\right)\right),\tau <t\leq T_{c}$$ (5)

where the term $\tau^{2}$ ( τ≪1 ) is neglected, $f_{IF}$ is the frequency of the IF signal, and $\varphi \left(t\right)$ is the phase of the IF signal, as drawn in Equation (6) (Alizadeh et al., 2019).

$$\varphi \left(t\right)=2\pi f_{c}\tau =4\pi \left(R_{0}+x\left(t\right)\right)/c$$ (6)

An FFT calculation is performed on each chirp sample of the radar-acquired IF signal, this FFT reveals range information, called the Range FFT. Each chirp (time axis) is sampled N times (range axis), and a distance block (range bin) is generated for each sample. Each Range FFT bin represents a specific distance, and this process is repeated for each chirp, so it will produce a Range FFT block of N×M dimensions, as displayed in Figure 2B where N is the number of FFT points and M is the number of chirps per frame. We need to locate the range bin where the target is located, and then extract the cardiopulmonary signals from that range bin.

In Figure 1A, chirp integration is performed for each Frame. The average value of n chirps in each Frame is used as the Chirp selected by this Frame. This operation assists to eliminate phase noise. Only one chirp is selected per frame, which reduces the sampling rate. It also reduces the amount of data processing calculations. We propose a lightweight algorithm for subsequent processing.

The proposed target state detection algorithm.

The proposed target state detection algorithm based on mixed-modal information is demonstrated in Figure 1B, which consists of three procedures: static clutter removal, confidence value calculation, and range bin variance. Static clutter removal is used to remove static background noise in the signal. The confidence value calculation is used to determine whether the target exists. The range bin variance is used to determine whether the target is stable or moving after detecting the presence of the target. The proposed algorithm can accurately detect three human target states.

1) Stable state is a stable human target state. It indicates that the range bin containing cardiopulmonary signal information with high SNR can be accurately selected;
2) Motion state is an intermediate state. It demonstrates that a human target can be detected. However, the human body has relatively large movements, with a large noise signal, and it is difficult to accurately extract cardiopulmonary signal information;
3) Unmanned state means that no human target is detected.

When the human target is in a stable state, the range bin is selected, and the cardiopulmonary signal with a higher SNR is extracted. Then we employ a simple FFT spectral analysis method to estimate the cardiorespiratory rate.

#### 2.1.1 Static clutter removal

In real environments, radar signals regularly contain a lot of noise. Radar receives reflected signals not only from the target but also from the environment and unwanted targets. Before locating potential live targets and extracting weak cardiopulmonary signal signals, static environmental noise needs to be removed from the data.

Then perform static clutter removal on the Range FFT information to remove more static points from the background. The model is expressed in Equation 8.

$$X_{m}^{\prime }\left[k\right]=\alpha X_{m}\left[k\right]+\left(1-\alpha \right)X_{m-1}^{\prime }\left[k\right]$$ (7)

$${{Y_{m}\left[k\right]=X}_{m}\left[k\right]-X}_{m}^{\prime }\left[k\right]$$ (8)

where m is the frame index, k is the range bin index, α is the filter coefficient, the larger the α value the stronger the clutter filtering effect, X is the Range FFT value, which is a complex number, $X^{\prime }$ is the calculated filtering parameter, and Y is the filtered Range FFT value.

**FIGURE 3:**
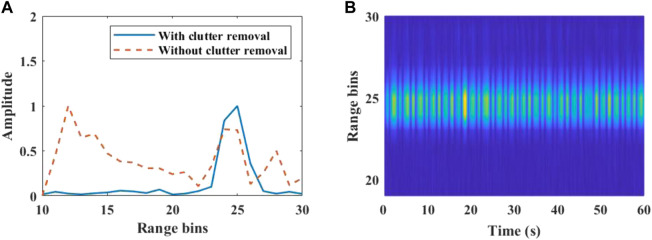
Target range bin localization. **(A)** Range FFT for a single frame. Some static clutter is suppressed after clutter removal. **(B)** Range profile across slow time.

Calculate the Range FFT amplitude of each frame after clutter removal, and obtain the range profile matrix containing the instantaneous distance information. Figure 3B manifests the range profile of the slow time dimension. The human target is located near the 25th range bin. The distance between the human and the radar is about 1.5 m.

#### 2.1.2 Confidence value calculation

Based on the set range bin scan range, the target range bin is searched from the range bin start index to the range bin end index. Constant False Alarm Rate (CFAR) (Kronauge and Rohling, 2013) is a technique for radar systems to detect the presence of target signals. We refer to this method to design a human target detection method based on confidence value. The range bin where the human target is located has much larger energy than other bins, and the range bin with the peak energy in the range profile is selected as the detection cell. The target confidence value is the energy ratio of detection cells to training units, as in Equation 9.

$$V\left(m\right)=P_{D}\left(m\right)/P_{T}\left(m\right)$$ (9)

$$P_{T}=\sum_{i=D-G-T}^{D-G}\left(P_{i}/T\right)+\sum_{j=D+G}^{D+G+T}\left(P_{j}/T\right)$$ (10)

where V is the confidence value, m is the frame index, $P_{D}$ is the peak energy of detection cells, $P_{T}$ is the average energy of training cells, D is the range bin index of peak energy, G is the number of single-side guard cells, and T is the number of single-side training cells.

**FIGURE 4:**
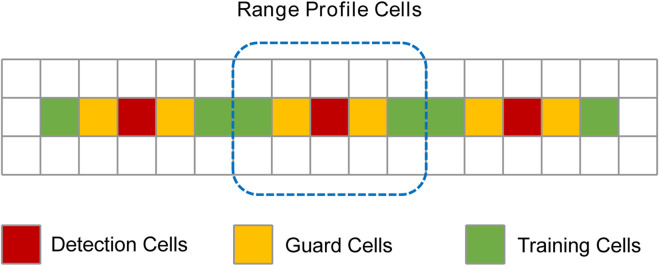
Confidence value calculation window.

Each window consists of cells as expressed in Figure 4, and the detection cells are the range bins of peak energy. Guard cells are on both sides of the detection cells and are used to prevent target signal energy from leaking into the training cells. When the target has a large random motion, some noise signal energy will enter the guard cells. Training cells are background noise energy cells. The number of guard cells and training cells is determined according to the environmental noise. Confidence calculations are performed on the range profile of each frame to detect targets. The edge range bin is processed in a circular supplementary manner, which can keep the same number of guard cells and training cells on both sides of the detection cells. Copy the range profile three times and merge it to generate a new matrix, and do calculations on the middle range profile. The cells that are not enough on the left are supplemented with the cells at the end of the first range profile, and the cells on the right are supplemented with the cells at the front of the third range profile.

There is an error in the determination of a single frame due to the influence of background noise and random body movements. A method of doing a cumulative calculation of V value data of *v* frames window is used, which can reduce the error, as in Equation 11.

$$V_{sum}\left(m\right)=\sum_{i=m}^{m+v}V\left(i\right)$$ (11)

**FIGURE 5:**
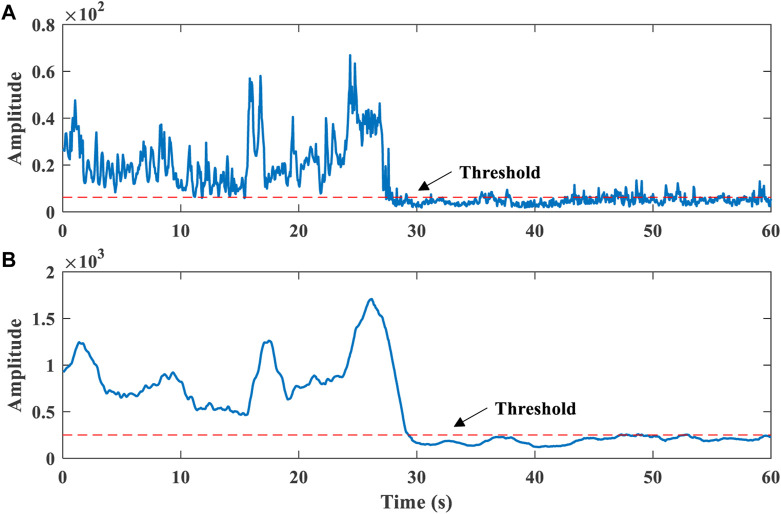
Effect of confidence value accumulation on human presence detection. **(A)** before the accumulation. **(B)** after the accumulation.

Figure 5 presents the effect of confidence value accumulation on human presence detection. The false detection rate decreases after accumulation.

In our work, we set a sliding window of 2 s (40 frames) and a step size of 1 frame for human presence detection, which provides real-time detection. The parameter of $V_{sum}$ greater than the set threshold means that the human target is detected. Otherwise, noise signals are detected and no human target signals are detected. The threshold is trained according to the background scene without people, which has a certain scene adaptive performance. To avoid target signals with small amplitudes being classified as noise, set the threshold lower.

#### 2.1.3 Range bin variance

In our proposed algorithm, the target should be in a stable state to extract the cardiopulmonary signal accurately. We introduce the variance $s^{2}$ to analyze the dispersion of the range bin in the time domain. After detecting a human target, it can be used to determine whether the target is in stable state or motion state.

$$s^{2}=\sum_{i=1}^{n}{\left(r_{i}-r\right)}^{2}/\left(n-1\right)$$ (12)

where $s^{2}$ is the range bin variance, n is the number of frames, $r_{i}$ represents the index of the range bin of the i^th frame, and r stands for the mean value of the range bin of the n frames.

**FIGURE 6:**
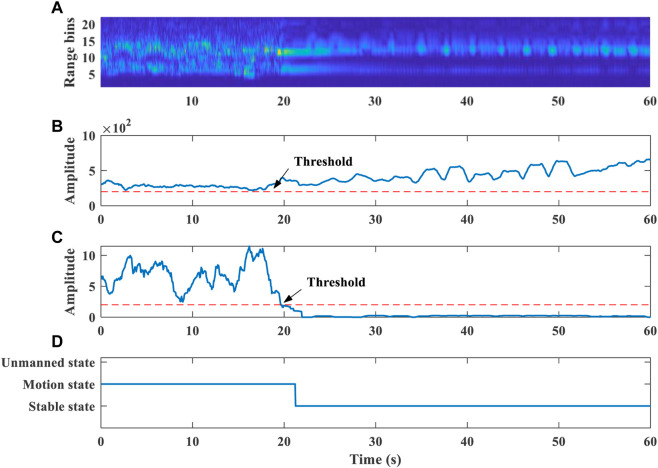
**(A)** Range profile across slow time. **(B)** Confidence value calculation results. **(C)** Range bin variance. **(D)** Target state detection.

Figure 6B shows that human objects are detected in the 60-s time. we set a sliding window of 2 s range bin variance buffer updated with 1 frame step size, which stores the state of the target every frame. After a human target is detected at each frame, the target state is determined by comparing the variance with a set threshold. The variance is less than the threshold, indicating that the human target is in stable state. If the variance is greater than the threshold, the human target is in motion state. Ideally, the human body should not move very much during the 1-frame period, so the range bin position and the current state will not change. When the proportion of any state in the buffer exceeds 80%, it is determined that the frame is in that state.

The time in the first 20 s of Figure 6C indicates that the range bins of the human body were detected to be cluttered, which means that the person is in a state of relatively large motion. The human body may have a large movement with excessive noise signals, which is not suitable for cardiopulmonary signal extraction. In the last 40 s, the human body is in a stable sitting state, which is stable state. We perform median smoothing of the 2 s window on the target range bin of stable state. Then select the range bin where the target is located, and extract the cardiopulmonary signal information. Our algorithm can make a target state decision within 2 s. The selection of range bins also has the same real-time performance.

### 2.2 Cardiopulmonary signal processing

Cardiopulmonary signals are processed using a sliding window of 20 s and a stepping update of 1 s to estimate respiration rate and heart rate. Cardiopulmonary signal processing consists of the following parts.

**FIGURE 7:**
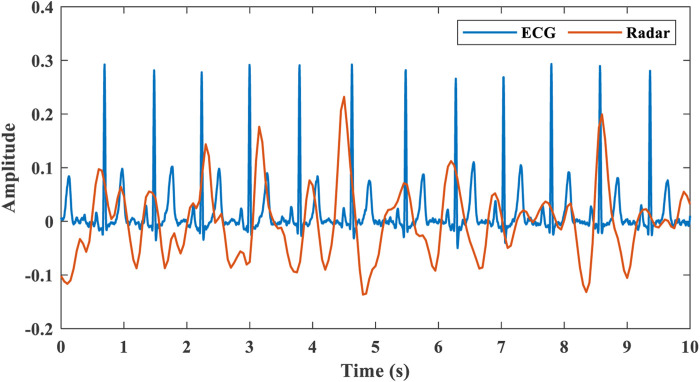
Comparison of extracted heartbeat signal by radar with ECG.

**FIGURE 8:**
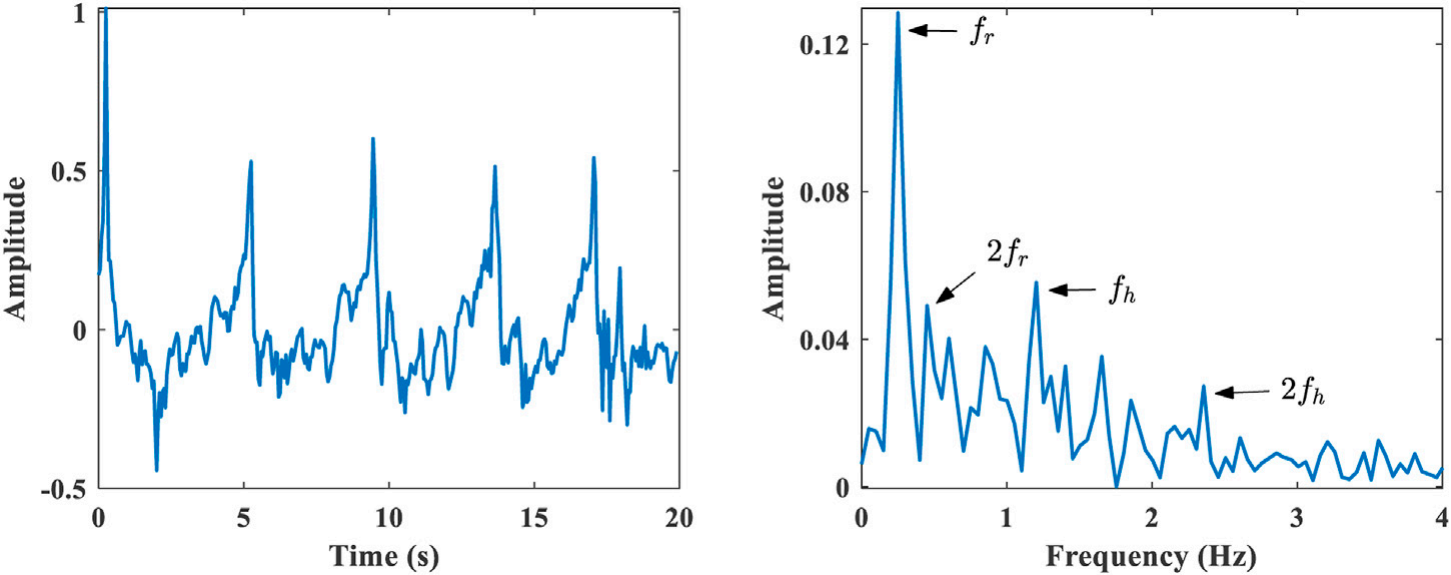
Extracted cardiopulmonary signal (after unwrapping and difference) and its spectrum.

1) Range bin selection: After using the proposed target state detection algorithm, select the range bin where the target is located. And extract the cardiopulmonary signal phase and amplitude information of the target.
2) Phase signal processing: Phase unwrapping recovers continuous phase changes. Then do the phase difference processing, which helps to eliminate the phase drift caused by the noise signal.
3) Separation of respiration and heartbeat: Respiration and heartbeat signals occupy different frequency ranges, which can be separated with appropriate band-pass filters. A fourth-order IIR cascaded biquad filter limits the frequency range to 0.1–0.6 Hz for respiration detection and 0.8–4 Hz for heartbeat detection. Figure 7 exhibits the comparison between the extracted heartbeat signal and the ECG collected by the wearable sensor.
4) Respiration rate estimation: FFT spectrum analysis is performed on the separated respiration signal. The respiration rate is selected by the largest peak within the spectrum. It can also be estimated by calculating the distance between the peaks of the time-domain waveform.
5) Heart rate estimation: FFT spectrum analysis is performed on the separated heartbeat signal. Respiratory harmonics will overlap with the spectrum of the heartbeat signal, which will affect the heart rate estimation, as shown in Figure 8. Where $f_{r}$ is the respiratory rate and $f_{h}$ is the heart rate. Extract N peaks (from large to small) in the frequency spectrum of the heartbeat signal, and eliminate the peaks corresponding to the respiratory harmonics. Then, the heart rate is selected by the largest peak within the spectrum.

The reference data collected by the wearable sensor is processed in the time domain, and the respiration rate and heart rate are estimated through a sliding window of the same size.

## 3 Experimental results

### 3.1 Experimental setup

The measured experimental scene is presented in Figure 2A. The FMCW radar has 1 Rx antenna and 2 Tx antennas. It has two receiving channel data, and we only select one of them for processing. The operating frequency of the radar is 23.5GHz–27.5 GHz. Each frame period is 50 ms, and each frame has 32 chips. The reference value data was collected using the Shimmer3 wearable wireless sensor, which is considered a reliable sensor for measuring vital rates, with a sampling frequency of 250 Hz.

There were 20 subjects tested. Each subject was fully informed of the experimental procedure and purpose and agreed to share measurement data anonymously. The patient experimental protocols of the First Affiliated Hospital of Nanjing Medical University were approved by the Ethics Committee (Reference code: 2020-SRFA-183). Each subject had a normal vital rate and rested for at least 10 min before the recording procedure. The radar was fixed in front of the subject at the same chest height to capture chest displacement. The subjects sat on a chair about 1.0 m away from the radar, and each subject was measured for 3 min.

### 3.2 Experimental results

**FIGURE 9:**
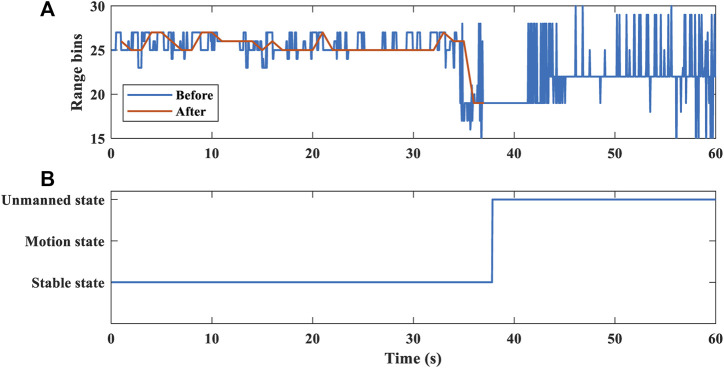
Human target localization. **(A)** Range bin selection before and after the proposed algorithm. **(B)** Target state detection.

To evaluate the work of the proposed method, we tested three states of the human target. The method can make an accurate decision on the target state within 2 s, as displayed in Figure 9A. In order to accurately extract the cardiopulmonary signal of the target, we test the person in a resting state and let the target leave the test area after a certain period. Figure 9B exhibits the results obtained before and after using the proposed method. Other objects and random background noise can interfere with range bin extraction. Before the proposed algorithm, we use the range bin selection method based on the maximum amplitude as a comparison method. Other objects and random background noise interfere with range bin extraction, so range bin extraction is accompanied by random jitter. After applying the proposed method, the smooth range bin information can be accurately obtained.

We performed spectral analysis using the FFT method on the extracted cardiopulmonary signal with a sliding window of 20 s and a step size of 1 s. The reported beats per minute (BPM) for respiration and heartbeat are averaged over 1-min segments. The mean absolute error (MAE) and root mean square error (RMSE) for all 5 subjects who participated in the experiments are compared with their related reference signals. They are represented as:

$$MAE=\frac{1}{n}\sum_{i=1}^{n}\left|y_{i}-x_{i}\right|=\frac{1}{n}\sum_{i=1}^{n}e_{i}$$ (13)

$$RMSE=\sqrt{\frac{1}{n}\sum_{i=1}^{n}{\left(y_{i}-x_{i}\right)}^{2}}$$ (14)

where $y_{i}$ is the radar-measured value, $x_{i}$ is the reference value and $e_{i}$ is their absolute error.

**FIGURE 10:**
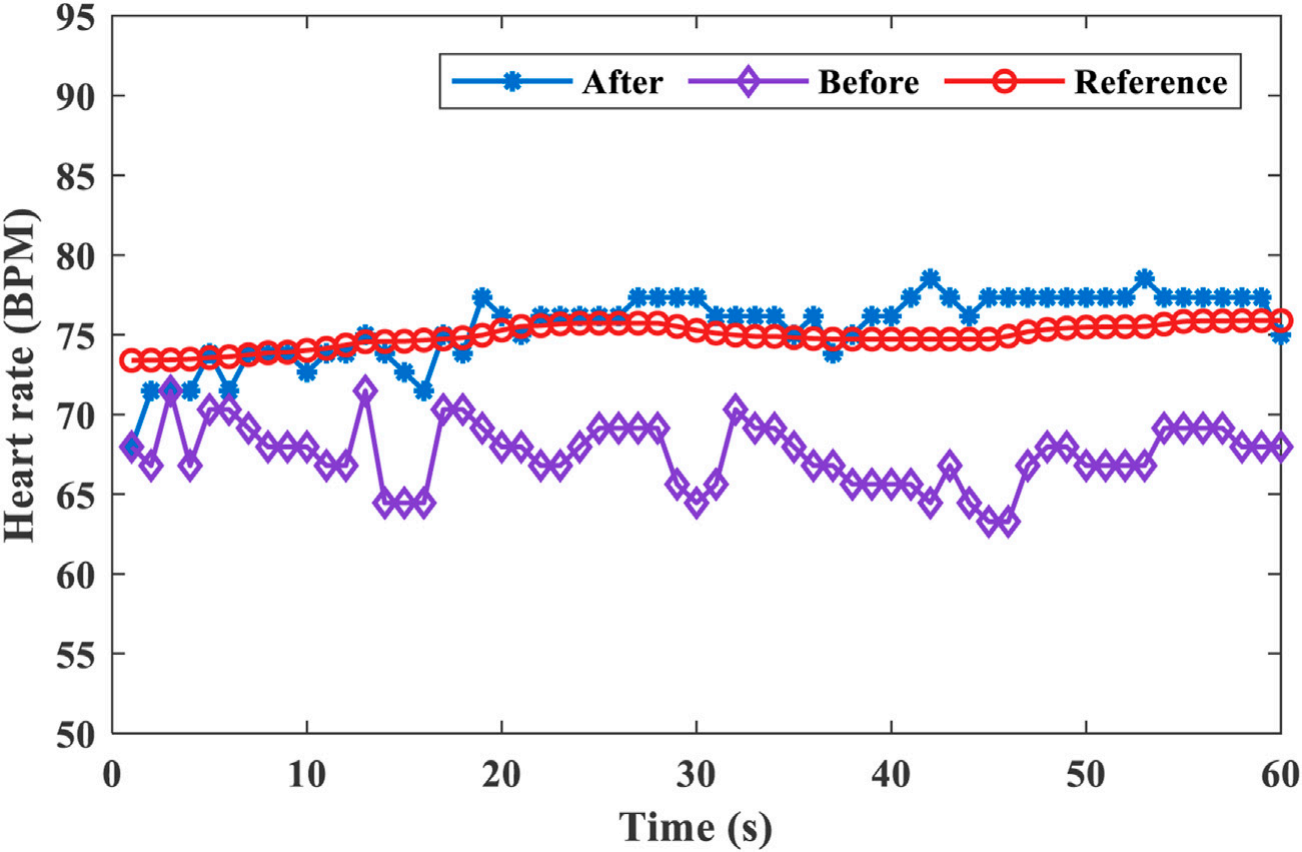
Comparison of heart rate estimates before and after applying the algorithm and the reference value.

One of the comparison results of heart rate estimation is shown in Figure 10. The average heart rate was 75.0 BPM estimated by the wearable sensor. Before applying the proposed method, the estimated average heart rate was 67.5 BPM, the overall MAE was 7.4 BPM, and the RMSE was 7.4 BPM. After applying the proposed method, the estimated average heart rate is 75.6 BPM, the overall MAE is 1.5 BPM, and the RMSE is 0.6 BPM. Due to the influence of small body random motion and background noise, the range bin selection will have a large error, which leads to an increase in the cardiopulmonary signal estimation error. After applying the proposed algorithm, the accuracy of range bin selection is improved, and the signal-to-noise ratio of the extracted cardiopulmonary signal is improved. It can be seen from Figure 10 that the estimated heart rate values are in good agreement with the reference heart rate after applying the proposed algorithm. The results show that the range bins selected by this method contain cardiopulmonary signals with higher SNR.

The results of the heart rate and respiration rate estimation errors before and after applying the proposed method are displayed in Table 1. The results were measured with 5 subjects with different situations in this experiment. The results give the average MAE and average RMSE of 1.0 BPM and 0.5 BPM for respiration rate estimation, and 1.9 BPM and 1.3 BPM for heart rate estimation after applying the proposed method. The results are all better than the comparison method. Before applying the algorithm, the heart rate estimation errors of targets A and D are both large. Because the background noise of the test environment and the random movement of the body are large, it interferes with the range bin selection of the human target. Therefore, the SNR of the extracted cardiopulmonary signal is low, and the estimation error of the cardiopulmonary rate is large.

**TABLE 1:** Measurement error results before and after applying the proposed algorithm. (MAE ± RMSE, BPM).

Subject	Respiration		Heartbeat	
	Before	After	Before	After
A	3.6 ± 3.1	1.5 ± 0.9	10.3 ± 10.3	2.8 ± 2.3
B	0.8 ± 0.3	0.8 ± 0.3	2.8 ± 1.1	2.7 ± 1.0
C	0.2 ± 0.1	0.2 ± 0.1	1.3 ± 1.0	1.2 ± 1.0
D	1.2 ± 0.9	1.1 ± 0.8	7.4 ± 7.4	1.5 ± 0.6
E	1.9 ± 1.6	1.3 ± 0.3	1.8 ± 1.7	1.5 ± 1.4
Average	1.5 ± 1.2	1.0 ± 0.5	4.7 ± 4.3	1.9 ± 1.3

Respiration rate accuracy was defined as the proportion of instances where the radar estimated respiration rate was within 2 BPM of the reference sensor, and heart rate accuracy was defined as within 5 BPM. Our experiments demonstrate an accuracy of 90.8% for respiration rate and 94.1% for heart rate. This result is better than the respiration rate accuracy of 83.7% and the heart rate accuracy of 70.7% reported by (Choi et al., 2020). Therefore, the proposed algorithm can accurately select the range bins containing cardiopulmonary signals with high SNR, and the accuracy performance is better in the respiration rate and heart rate estimation.

## 4 Conclusion

In this paper, we propose a target state detection algorithm based on a mixed-modal threshold for target state detection and cardiopulmonary signal range bin selection. First, the algorithm can accurately detect the target state in different scenarios in real time. It can avoid range bins with larger noise signals and select range bins containing cardiopulmonary signals with high SNR, which improves the accuracy of respiration rate and heart rate estimation. In the experimental results, we analyze the radar recordings of 5 subjects in different scenarios and different motion states. We select range bins and extract cardiopulmonary signals by the proposed algorithm, and estimate respiration rate and heart rate by the FFT method. The results demonstrate that the proposed algorithm has better performance than traditional range bin selection methods in terms of cardiopulmonary signal estimation. Furthermore, it performs well on low sampling rate data. it requires less computing power and high performance for real-time evaluation, which is of great value for forming a lightweight embedded system on a low-cost low-power radar chip.

Nevertheless, this work also has some limitations. During the experiment, the subjects were required to remain relatively still. Because the vibration noise of the environment with a frequency close to the heart rate will interfere with the heart rate estimation. In future research, we intend to conduct embedded system testing experiments in various practical situations such as home care, medical monitoring, driver monitoring systems, etc.

## Data Availability

The original contributions presented in the study are included in the article, further inquiries can be directed to the corresponding authors.
